# Transcranial Direct Current Stimulation Altered Voluntary Cooperative Norms Compliance Under Equal Decision-Making Power

**DOI:** 10.3389/fnhum.2018.00265

**Published:** 2018-07-03

**Authors:** Jianbiao Li, Xiaoli Liu, Xile Yin, Shuaiqi Li, Guangrong Wang, Xiaofei Niu, Chengkang Zhu

**Affiliations:** ^1^China Academy of Corporate Governance, Reinhard Selten Laboratory, Business School, Nankai University, Tianjin, China; ^2^Nankai University Binhai College, Tianjin, China; ^3^Neural Decision Science Laboratory, Weifang University, Weifang, China

**Keywords:** voluntary cooperative norm, norm compliance, equal decision-making power, transcranial direct current stimulation, right dorsolateral prefrontal cortex

## Abstract

Social norms play an essential role in human interactions and the development of the evolution of human history. Extensive studies corroborate that compliance with social norms typically requires a punishment threat as almost always specific individuals have self-interests that tempt them to violate the norm. Neural imaging studies demonstrate that lateral orbitofrontal cortex (LOFC) and right dorsolateral prefrontal cortex (rDLPFC) are activated when individuals decide to increase social norm compliance when punishment is possible. Moreover, rDLPFC is affirmed to be involved in social norm compliance with or without external punishment threats in a series of transcranial direct current stimulation (tDCS) research. However, these neuroscience studies are based on the ultimatum game (UG) in which the decision-making power between the proposer and the responder is unequal, and no studies support the causal relationship between rDLPFC and voluntary cooperative norms compliance among the equal decision-making power of subjects. Whether modulating the excitability of rDLPFC, which plays a role in norm compliance, alters the extent of compliance with voluntary cooperative norms under equal decision-making power and how norms from different types with asymmetric endowment influence compliance remain unknown. The present study aimed to provide evidence of a direct link between the neural and behavioral results through the application of tDCS over rDLPFC on compliance with voluntary cooperative norms under equal decision-making power. Results verified that activating rDLPFC altered voluntary cooperative norms compliance of all our participants and significant effect over different initial endowments was observed. The role of norm.own and norm.other in compliance was changed in the anodal treatment. Findings validate that enhancing the excitability of the rDLPFC using tDCS leads to high compliance in voluntary cooperation and this effect is specific to equal decision-making power rather than unequal decision-making power.

## Introduction

Social norm compliance indicates people rely on norms to guide their behavior to establish social relationships (Spitzer et al., 2007; Ruff et al., 2013). In recent years, social norm compliance is explained as a black box meant to capture a few of the influences of social environment on individuals’ decisions (Andreoni and Douglas Bernheim, 2007; Fishbein and Icek, 2010; Schram and Charness, 2015). Investigations of social norm compliance usually focus on either social norms can be enforced by threatening norm violators in the framework with sanctions options like ultimatum game (UG; Forsythe et al., 1994; Fehr and Gächter, 2002; Nelissen and Mulder, 2013; Eriksson et al., 2017), or it can be voluntary maintained in the framework such as public goods game (PG) and prisoner dilemma game (Nese and Sbriglia, 2009; Reuben and Riedl, 2013; Amegashie, 2016; Chaudhuri et al., 2016; Realpe-Gómez et al., 2018).

A long stream of behavioral experiments on UG affirms that norm compliance can be maintained by sanctioning threats (Sober and Wilson, 1998; Gintis et al., 2003; Mathew and Boyd, 2011; Eriksson et al., 2017). For example, Mendoza et al. (2014) validated that social norm concerns led to punishment responses in the UG. Similarly, Nelissen and Mulder (2013) showed that punishment induced compliance with norms for cooperation in UG. These behavioral studies validated that sanctions plays an essential role in social cooperation by generating appropriate behavioral responses for norm compliance. Meanwhile, experiments in PG find evidences of voluntary norm compliance. For example, subjects on average contribute 40% to 60% of their total endowment in public goods games, although they can make zero contribution to maximize their monetary payoff (Chaudhuri and Paichayontvijit, 2006). These results suggest that subjects are not only care about their own payoff, but also show motivations to follow social norms which emphasize voluntary cooperation (Nese and Sbriglia, 2009; Reuben and Riedl, 2013).

The human brain is proposed to have developed neural processes that support norm compliance (Lotze et al., 2007; Montague and Lohrenz, 2007; Spitzer et al., 2007; Baumgartner et al., 2011). Moreover, the rapidly growing field of decision neuroscience has investigated how brain areas such as dorsolateral prefrontal cortex (DLPFC) affect human norm compliance (Forsythe et al., 1994; Pillutla and Murnighan, 1996; Fehr and Gächter, 2002; Raine and Yang, 2006; Baumgartner et al., 2011). For example, through functional magnetic resonance imaging technology, Sanfey et al. (2003) proved that DLPFC were associated with social norm compliance in the UG. Similarly, Spitzer et al. (2007) found that lateral orbitofrontal cortex (LOFC) and right DLPFC (rDLPFC) were activated when individuals decided to increase social norm compliance when punishment was possible in the UG. In order to investigate the casual relation between rDLPFC and norm compliance, Knoch et al. (2006) used transcranial magnetic stimulation in UG and found subjects whose rDLPFC was disrupted exhibit a much higher acceptance rate than those in the other two control groups. Besides, Ruff et al. (2013) proved that social norm compliance was changed while the activity of rDLPFC was altered by transcranial direct current stimulation (tDCS). Combining functional magnetic resonance imaging and transcranial magnetic stimulation, Baumgartner et al. (2011) also examined rDLPFC was causally involved in norm compliance in UG. Therefore, rDLPFC plays an important role in social norm compliance.

Previous neural studies are based on the UG and rarely consider roles of rDLPFC in social norm compliance under PG. In UG the proposer is confronted with the sanction threat of the responder, making their cooperation involuntary. On the contrary, participants in PG make their own contributions without any concerns of sanctions, that is, they can decide whether to cooperate voluntarily. Meanwhile, the decision-making power of participants is unequal: the proposer is entitled with power to allocate endowments between himself and the responder, while the responder can only accept or reject the allocation. Therefore, the distinctions of decision order and strategic set between them lead to the inequality of their decision-making power. Different from these studies using the UG, the decision-making power of participants in PG is equal. Therefore, PG is appropriate to study human voluntary cooperation behavior under equal decision-making power (Fehr and Schmidt, 1999; Kurzban and Houser, 2001; Spiller et al., 2016).

We aim to directly provide this missing piece of previous research by examining whether modulating the excitability of rDLPFC by tDCS alters cooperative norm compliance in PG, which includes two types of participants with asymmetric endowment (RICH and POOR). We randomly divided participants into three treatments, in which neural excitability in rDLPFC was enhanced with anodal tDCS, reduced with cathodal tDCS or left unaltered by sham tDCS as control for possible non-neural effects of stimulation. To the extent we know, this is the first study to explore the role of rDLPFC in voluntary cooperative norm compliance by means of tDCS.

According to previous evidence (Sanfey et al., 2003, 2014; Knoch et al., 2006; Spitzer et al., 2007; Baumgartner et al., 2011; Ruff et al., 2013), enhancing/suppressing rDLPFC response increased/decreased the level of norm compliance. We assume that if anodal/cathodal of tDCS was applied to increase/decrease the activities of rDLPFC, participants would alter their contribution in the PG, that is, their norm compliance would improve/deteriorate. Further, we investigate whether tDCS effect on the norm compliance is different between RICH and POOR types.

## Materials and Methods

### Participants

Eighty-four healthy college students were recruited to participate in our experiment. One participant in the anodal treatment felt discomfort with stimulation, and we stopped the experiment. Overall, eighty-three subjects (42 men and 41 women; mean age = 24.04, SD = 2.75, ranging from 20 to 30 years old) were kept in the sample. All these participants were right-handed without *ex ante* knowledge of tDCS or public games. All of them had no history of psychiatric illness or neurological disorders. The participants were randomly assigned to the sham treatment (*n* = 28, 12 males, mean age = 23.39, SD = 2.67), cathodal treatment (*n* = 28, 12 males, mean age = 24.61, SD = 2.56) and anodal treatment (*n* = 27, 18 males, mean age = 24.11, SD = 2.98). Written informed consent was obtained from each participant prior to the study. The experiment was performed in accordance with the Declaration of Helsinki and was approved by the Ethics Committee of Reinhard Selten Lab of Nankai University. All these 83 participants did not report any adverse side effects concerning pain on the scalp or headaches after the experiment.

### Transcranial Direct Current Stimulation

**Figure 1:**
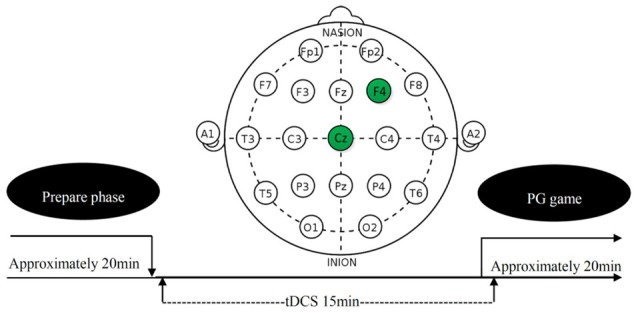
Schematic representation of the experimental design.

tDCS involves the application of a weak direct current to the scalp via two saline-soaked surface sponge electrodes. It can increase or decrease neural excitability in the stimulated region, depending on the polarity of the current flow, anodal tDCS increases neural excitability, whereas cathodal tDCS decreases it (Nitsche and Paulus, 2000; Batsikadze et al., 2013; Jamil et al., 2017). On the basis of this finding and rDLPFC’s general role in the norm compliance (Miller and Cohen, 2001; Aron et al., 2014; Spitzer et al., 2007; Baumgartner et al., 2011; Ruff et al., 2013), we randomly sorted participants into three treatments (anodal, cathodal and sham). In anodal treatment, the neural excitability in rDLPFC was enhanced. In cathodal treatment, the neural excitability in rDLPFC was reduced. In the sham treatment, the neural excitability in rDLPFC was left unaltered as control for possible non-neural effects of the other two treatments. The current was constant (1.0 mA) with 15 s of ramp up and down (Ambrus et al., 2012; Meesen et al., 2014; Li et al., 2017; Wang et al., 2017). The tDCS, delivered by a battery-driven stimulator (Neuro Conn, Germany), was applied using a set of standard 5 × 7 cm electrodes fixed by rubber straps. These standard electrodes were chosen over custom. For the subjects receiving the tDCS, the anodal/cathodal electrode was placed over the rDLPFC (F4 in Figure 1; Sanfey et al., 2003; Spitzer et al., 2007; Ruff et al., 2013), and the reference electrode (cathode for anodal tDCS and anode for cathodal tDCS) was positioned over the vertex (CZ in Figure 1). After 15 min of stimulation, the participants were then asked to complete the task (see in the “Task and Procedure” section). The experimental design was presented in Figure 1. The procedures in the sham treatment were as the same as anodal and cathodal treatments, except that the current stopped after the first 30 s. Civai et al. (2015) and Willis et al. (2015) had proved that the 30 s stimulation in the sham condition could mimic the itching sensation of the real stimulation without significantly changing the activity of cortex.

### Task and Procedure

The task in this article was similar to those conducted by Spiller et al. (2016). Compared to their design, participants in this experiment received tDCS 15 min before they participated in the experimental task. The participants engaged via computer terminals in anonymous social interactions. Their decision was based on real financial consequences. They used game dollar (G$) as the unit of payoff in the experiment. All the G$ they earned were exchanged to Chinese Yuan (RMB) after the experiment. The exchange ratio was 1 G$:1.5 RMB. They were paid in cash. Each participant was paid according to their own offers and decisions of their partners. On average, each participant received approximately 50 RMB (7–8 dollars).

Participants were randomly divided into groups of four. Then, they were assigned the types. Two of them were RICH players with 35 G$(A1, A2) and the others in groups of four were POOR players with 23 G$(B1, B2). Participants were informed their types at the beginning of each period.

In the experiment, participants were asked to answer the questions: “What do you think is the “right” contribution? If they were RICH players (A1 and A2) and POOR players (B1 and B2), respectively”. After that, they played a linear PG with asymmetric endowment. Participant *i* faced the following payoff function: $\pi_{i}={\text{X}}_{i}-x_{i}+0.6\sum_{i=1}^{4}x_{i}$, where X_*i*_ was her endowment, *x*_*i*_ was her contribution to the PG, and $\sum_{i=1}^{4}x_{i}$ was the sum of all participants’ contributions in the same group.

A total of 16 periods were conducted. In each period, the endowments were started from the initial situation and the types (RICH and POOR) of participants were reassigned. The participants were randomly assigned as RICH types in eight of sixteen periods and POOR types in other eight periods. Participants had not any feedback about contribution and payoff. They were not informed how many periods they would play either. They had no additional information except their own type and types of the other three participants in their group.

### Statistical Analysis

There are two types of players, namely: (1) RICH (35G$, A1 and A2); and (2) POOR (23G$, B1 and B2). For each type, there are four pairs of players: (1) RICH for RICH (indicates RICH players to the question for RICH players); (2) POOR for RICH (indicates POOR players to the question for RICH players); (3) RICH for POOR (indicates RICH players to the question for POOR players); and (4) POOR for POOR (indicates POOR players to the question for POOR players). The level of voluntary cooperative norm was assessed using the “right” contribution asked during the experiment (What do you think is the “right” contribution?). Three types of voluntary cooperative norm were tested: (1) norm.all (indicates one’s standard about “right” contribution of all participants); (2) norm.own (indicates one’s standard about “right” contribution of same initial endowment, i.e., RICH for RICH and POOR for POOR); and (3) norm.other (indicates one’s standard “right” contribution of different endowment, i.e., RICH for POOR and POOR for RICH). The level of voluntary cooperative norm compliance was assessed using participants’ real contribution in the PG. Three types of voluntary cooperative norm compliance were tested: (1) compliance.ALL (indicates voluntary cooperative norm compliance of all players); (2) compliance.RICH (indicates voluntary cooperative norm compliance of RICH player); and (3) compliance.POOR (indicates voluntary cooperative norm compliance of POOR player). Three treatments were formed: (1) anodal; (2) sham; and (3) cathodal.

We used STATA software (version 12) to analyze data of this experiment. To examine the influence of types and tDCS treatments on the voluntary cooperative norm compliance (compliance.ALL), we performed two-way ANOVA: 2 (types of players: RICH and POOR) × 3 (tDCS treatments: anodal, sham and cathodal). In order to compare the mean of compliance.RICH and compliance.POOR in three treatments, one-way ANOVA was then performed, respectively. In addition, the difference between the whole compliance (compliance.ALL) and norm (norm.all) in three treatments were evaluated using paired sample *t*-test (i.e., paired *t*-test). Moreover, we ran ordinary least squares (OLS) regression to examine how norms from different types (norm.own and norm.other) influence participants’ compliance behavior (compliance.ALL).

## Results

### Effect of tDCS Over rDLPFC on Norm Compliance

We performed two-way ANOVA for compliance.ALL with the player type (RICH and POOR) as a within-subject factor and the treatment (anodal, cathodal and sham) as a between-subject factor. Significant main effects of treatment (*F*_(2,162)_ = 29.07, *p* < 0.001) and player type (*F*_(1,162)_ = 28.26, *p* < 0.001) were noted. Importantly, a significant interactive effect of stimulation type and player type was found (*F*_(2,160)_ = 3.82, *p* = 0.024). These results are in line with the previous study (Ruff et al., 2013), the two active brain stimulation conditions changed voluntary cooperative norms compliance relative to the sham condition, and our hypothesis was verified.

**Figure 2:**
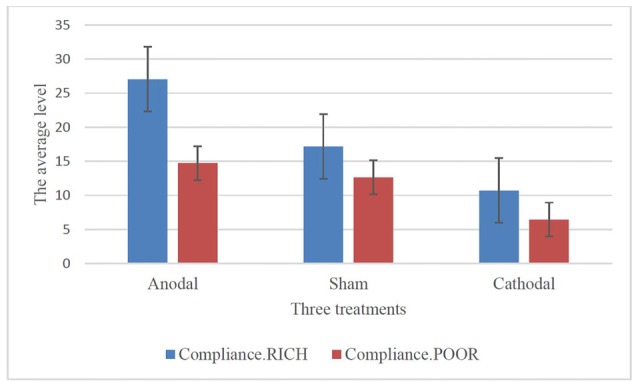
Compliance.RICH and compliance.POOR in three treatments.

To further confirm this conclusion, one-way ANOVA was used to analyze the difference among the voluntary cooperative norm compliance (compliance.RICH and compliance.POOR) of the three treatments, respectively. According to Table 1 and Figure 2, significant differences were observed in the compliance.RICH of the three treatments (*F*_(2,80)_ = 20.87, *p* < 0.001). Similarly, significant differences were observed in the compliance.POOR of the three treatments (*F*_(2,80)_ = 10.46, *p* < 0.001). It meant that both types of participants were sensitive to the tDCS. In addition, the current data in Table 1 showed that the average level of compliance.RICH in the anodal, sham and cathodal treatments were 27.04 (SD = 11.42), 17.18 (SD = 8.47) and 10.71 (SD = 8.10) and the average level of compliance.POOR in the anodal, sham and cathodal treatments were 14.74 (SD = 9.76), 12.64 (SD = 6.40) and 6.11 (SD = 6.10), respectively. From sham to stimulation, the ratios of compliance.RICH and compliance.POOR increased by 57.39% and 16.46% in the anodal treatment, and the matching ratios were attenuated by 37.66%, and 51.66% in the cathodal treatment, respectively. The influence of rDLPFC activity on voluntary cooperative norm compliance was different between two types of participants (RICH and POOR). Overall, these results verified that activating the rDLPFC altered the voluntary cooperative norms compliance of all our participants and that significant effect over different initial endowments was observed.

**Table 1:** Results of one-way ANOVA.

Variables	tDCS	Mean	Std. Dev.	One-way ANOVA
	Anodal	27.04	11.42	
Compliance.RICH	Sham	17.18	8.47	*F*_(2,80)_ = 20.87, *p* < 0.001
	Cathodal	10.71	8.10	
	Anodal	14.74	9.76	
Compliance.POOR	Sham	12.64	6.40	*F*_(2,80)_ = 10.46, *p* < 0.001
	Cathodal	6.11	6.10	

**Figure 3:**
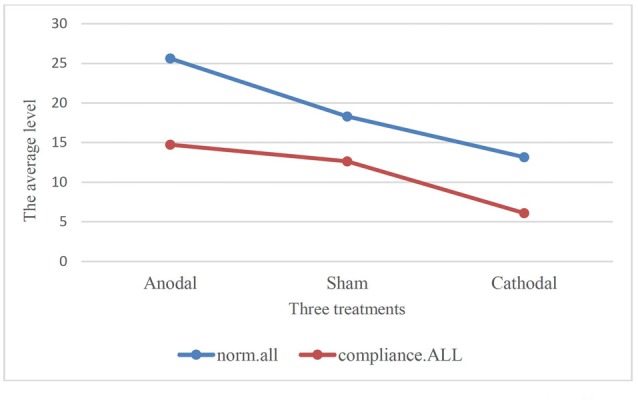
Comparison of norm.all and compliance.ALL in three treatments.

Moreover, we examined the relation of compliance.ALL and norm.all with paired comparison among all participants under three treatments. We conducted paired *t*-test. One stable main effect was found: subjects’ compliance in the experiment (compliance.ALL) was significantly lower than the cooperative norm (norm.all) they report (18.95 (SD = 9.11) > 11.12 (SD = 8.09); *t* = 14.513, *p* < 0.001, paired *t*-test). The relationship also existed in the three treatments. According to Table 2 and Figure 3, the results corroborated that the average of norm.all was significantly higher than compliance.ALL in the three treatments (anodal treatment 25.62 (SD = 7.89) > 14.74 (SD = 9.63); *t* = 8.089, *p* < 0.001, paired *t*-test, sham treatment 18.31 (SD = 6.73) > 12.64 (SD = 6.31); *t* = 7.340, *p* < 0.001, paired *t*-test, cathodal treatment 13.17 (SD = 8.04) > 6.11 (SD = 5.05); *t* = 10.410, *p* < 0.001, paired *t*-test). Figure 3 showed that the norm.all line was always above the compliance.all line. This was consistent with the aforementioned results, and the hypothesis was verified again.

**Table 2:** Results of paired *t*-test.

Treatments	Paired variables	Mean	Std. Dev.	Paired *t*-test
Anodal	norm.all	25.62	7.89	*t* = 8.089, *p* < 0.001
	compliance.ALL	14.74	9.63	
Sham	norm.all	18.31	6.73	*t* = 7.340, *p* < 0.001
	compliance.ALL	12.64	6.31	
Cathodal	norm.all	13.17	8.04	*t* = 10.410, *p* < 0.001
	compliance.ALL	6.11	5.05

### OLS Regression Results of Norm Compliance

From sham to stimulation (anodal and cathodal), the ratios of individual compliance increased by 55.30% (RICH to RICH), 41.13% (RICH to POOR), 39.27% (POOR to RICH) and 34.71% (POOR to POOR) in the anodal treatment, and the matching ratios were attenuated by 34.09%, 34.41%, 30.15% and 35.17% in the cathodal treatment, respectively. We may conjecture that the different compliance behaviors are influenced by the norm of different types (norm.own and norm.other). The results from our regression analysis support this view (see Table 3).

**Table 3:** Ordinary least squares (OLS) regression results.

Regression	Anodal			Sham			Cathodal		
Variable	Reg. 1	Reg. 2	Reg. 3	Reg. 1	Reg. 2	Reg. 3	Reg. 1	Reg. 2	Reg. 3
Intercept	12.173**	37.806***	30.101***	6.802**	16.973***	8.186**	2.738*	6.178**	3.517*
	(5.079)	(5.903)	(8.313)	(2.654)	(3.225)	(4.052)	(1.554)	(1.865)	(1.762)
norm.own	0.348*		0.243	0.443**		0.438**	0.427***		0.488***
	(0.192)		(0.186)	(0.135)		(0.137)	(0.099)		(0.119)
norm.other		−0.647**	−0.585**		−0.113	−0.070		0.171	−0.122
		(0.218)	(0.221)		(0.502)	(0.154)		(0.123)	(0.129)
*R*^2^	0.059	0.145	0.173	0.166	0.008	0.169	0.256	0.034	0.268

Table 3 showed the results of regression analysis for two variables separately (Reg. 1 and Reg. 2) and compounded of the two (Reg. 3). Dependent variable: compliance.ALL (participants’ contributions in public goods game, PG); Numbers in parentheses were standard deviation; R2, coefficient of determination, was a statistical measure of how well the regression predictions approximate the real data points. *Significant level: p < 0.1; ***Significant level: p < 0.05; **Significant level: p < 0.01.

Regressions using “norm.own” and “norm.other” were conducted. This setting allowed us to enhance above result for the norm of different types. Table 3 showed that “norm.own” had a positive effect on compliance behavior and that “norm.other” had a negative effect except the Reg. 2 of cathodal treatment. Especially, in Reg. 1 and Reg. 3, the coefficient of “norm.own” in anodal, sham, and cathodal was similar and significantly positive. It meant that participants identified strongly with the norm.own and tended to make their compliance of the norm from same type. As Akerlof and Kranton (2010) suggested, one’s acts would in accord with the social norm in order to be a respected group member. These results suggest that the “norm.own” is the core promotion factor when subjects perform voluntary cooperative norm compliance.

Further, Table 3 also showed that the two active brain stimulation conditions (anodal and cathodal) changed the coefficients. Anodal stimulation reduced the influence of “norm.own” (0.348* < 0.443**, 0.243 < 0.438***) and improved the influence of “norm.other” (−0.647** < −0.113, −0.585** < −0.070) compared with the sham treatment, whereas cathodal stimulation had no significant influence on “norm.own” and “norm.other”. Therefore, the anodal stimulation changed the roles of the norms from different types (“norm.own” and “norm.other”). Unfortunately, no similar effect on cathodal stimulation was found. This situation may be normal because the dual-polarity effect (anodal-excitation and cathodal-inhibition effects, AeCi) had not been observed in all tDCS studies (Jacobson et al., 2012).

Further analysis of the available data, norm.own reflecting the rule “Do I behave according to others who like me?”, and norm.other reflecting the rule “Do I behave according to others who do not like me?” subjects, therefore, are more likely to behave according to the same type rather than the different types. The subjects identified strongly with the norm of the same type and tend to make their compliance. For the norm of different type, it is the exact opposite, that is, the subjects identified weakly and rarely follow. The results concur with identity theory (Stryker, 1968, 1987; Turner, 1978; Charness et al., 2007) and demonstrate a role for social identity.

## Discussion

In our study, anodal tDCS improved the voluntary cooperative norms compliance of the participants compared with the sham treatment, whereas cathodal tDCS deteriorated the voluntary cooperative norms compliance of the participants. This phenomenon existed between RICH player (35 G$) and POOR player (23G$). However, the degree of change was different. The anodal rather than cathodal stimulation changed the roles of the norms of different types (“norm.own” and “norm.other”). Our results supported a causal link between rDLPFC functioning and the voluntary cooperative norms compliance process, especially norm of different type. We found new evidence using tDCS through a stimulation applied over rDLPFC in voluntary cooperation under equal decision-making power compared to previous fMRI and tDCS studies (Spitzer et al., 2007; Ruff et al., 2013; Sanfey et al., 2014).

Our findings are consistent with prior studies, which corroborate that LPFC is essential for social norm compliance (Sanfey et al., 2003, 2014; Spitzer et al., 2007; Ruff et al., 2013). Social norm compliance contains sanction induced compliance and voluntary compliance. The role of sanctions in social norm compliance is supported by previous neuropsychological studies (Spitzer et al., 2007; Baumgartner et al., 2011; Ruff et al., 2013; Sanfey et al., 2014). A strong positive correlation between the increase in norm compliance and brain activation in rDLPFC has been found under the threat of social punishment (Spitzer et al., 2007; Baumgartner et al., 2011; Ruff et al., 2013; Sanfey et al., 2014). However, little research on voluntary cooperative norm compliance exist, and our research complements this area. Our finding supports the idea that rDLPFC is a crucial brain region involved in the process of social norm compliance not only under the enforcement of sanctions based on the UG but also under voluntary cooperation based on the PG.

Results in previous studies involving the UG affirmed that the participants used a fairness norm of “equity”. It means that the optimal decision would be to split the pot of money equally between proposers and responders (Ruff et al., 2013). The “equity” norm results from inequality aversion (Fehr and Schmidt, 1999; Bolton and Ockenfels, 2000), which assumes that people are intrinsically adverse to inequality. From the perspective of these dimensions, most people care about ensuring that others receive similar payoffs even without sanctions, as consequence of intrinsic motivation (Schram and Charness, 2015). On the basis of the idea above, the norm compliance mechanism in voluntary cooperative norms is similar.

However, previous norm compliance literature does not typically consider the relationship between the perception of norms and norm compliance. In this research, the role of norm.own and norm.other in norm compliance was changed through tDCS on rDLPFC, although the change was insignificant in the cathodal treatment. The effect of rDLPFC on cooperative norm compliance is speculated to may be different based on the correlation between the neural excitability of this brain region and the perception of the norms. In the anodal treatment, the subjects may increase in sensitivity to the perception of the norms of their own type and other type as rDLPFC activity increases. On the contrary, in the cathodal treatment, the subjects may decrease in sensitivity to the perception of the norms of their own type and other type as rDLPFC activity decreases. Their behaviors show that tracing the source of the norm is difficult, even show approve norm from other type.

This research can be expanded as follows. First, behavioral and neurophysiological evidence demonstrating other factors may also matter in social cooperation, such as the perceptions of decision-making power and social network sites (Fehr and Schmidt, 1999; Charness et al., 2007; Diga and Kelleher, 2009; Chang et al., 2011; Chang and Sanfey, 2013; Sanfey et al., 2014). Examining the specificity of these effects might be included in the future investigations of cooperative norm compliance. Further, do rDLPFC or other brain areas interact with these factors in affecting norm compliance? A possible extension for future research is to identify the other potential mediating variables of the modulatory effect of tDCS on voluntary cooperative norms compliance. Second, our results verify that the neural excitability of rDLPFC may lead to different norm perceptions (norm perception of the same type and norm perception of different type). In the future, investigating how the neural excitability of brain generates or modulates norm perception under different conditions may bring new changes in deep psychological and neural insights.

## Conclusion

Enhancing/suppressing activity in rDLPFC via anodal/cathodal stimulation increased/decreased voluntary cooperative norms compliance with equal decision-making power. Stimulation affects the compliance of the cooperative norm, suggesting that rDLPFC is necessary for norm-compliant behavior free from sanctions. In this study, all the subjects were sensitive to their own initial endowments. That is, the subjects identified strongly with the norm of same type and tend to comply. Contrarily, the subjects identified weakly with the norm of different type and rarely follow. This finding is a promising step to understand how neurobiological mechanisms relate to voluntary cooperative norms compliance.

## Author Contributions

JL and XL designed experiment; wrote the manuscript. XL, XN and CZ performed experiment. XL and GW analyzed data. XL and XY drew figures. JL, XL, XY and SL revised the manuscript and all authors finally approved the version to be published.

## Conflict of Interest Statement

The authors declare that the research was conducted in the absence of any commercial or financial relationships that could be construed as a potential conflict of interest. The reviewer BG-M and handling Editor declared their shared affiliation.
